# Conventional and Novel Technologies in the Production of Dairy Bioactive Peptides

**DOI:** 10.3389/fnut.2022.780151

**Published:** 2022-05-26

**Authors:** Mian Anjum Murtaza, Shafeeqa Irfan, Iram Hafiz, Muhammad Modassar A. N. Ranjha, Abdul Rahaman, Mian Shamas Murtaza, Salam A. Ibrahim, Shahida Anusha Siddiqui

**Affiliations:** ^1^Institute of Food Science and Nutrition, University of Sargodha, Sargodha, Pakistan; ^2^Institute of Chemistry, University of Sargodha, Sargodha, Pakistan; ^3^School of Food Science and Engineering, South China University of Technology, Guangzhou, China; ^4^Department of Food Science and Technology, Muhammad Nawaz Shareef (MNS) University of Agriculture, Multan, Pakistan; ^5^Food Microbiology and Biotechnology Laboratory, North Carolina Agricultural and Technical State University, Greensboro, NC, United States; ^6^Campus Straubing for Biotechnology and Sustainability, Technical University of Munich, Straubing, Germany; ^7^German Institute of Food Technologies (DIL e.V.), Quakenbrück, Germany

**Keywords:** dairy proteins, bioactive peptides production, green technologies, ultrasound-assisted extraction, fermentation, enzymatic hydrolysis

## Abstract

**Background:**

In recent years, researchers have focused on functional ingredients, functional foods, and nutraceuticals due to the rapidly increasing interest in bioactive components, especially in bioactive peptides. Dairy proteins are a rich and balanced source of amino acids and their derived bioactive peptides, which possess biological and physiological properties. In the dairy industry, microbial fermentation and enzymatic hydrolysis are promising methods for producing bioactive peptides because of their rapid efficiency, and mild reaction conditions. However, these methods utilize less raw material, take long reaction time, result in low yields, and low activity products when used alone, which pose industry to seek for novel methods as pretreatments to increase the yield of bioactive peptides.

**Scope and Approach:**

This review emphasizes the production of peptides from the dairy proteins and discusses the potential use of novel technologies as pretreatments to conventional methods of bioactive peptides production from dairy proteins, including the mechanisms of novel technologies along with respective examples of use, advantages, limitations, and challenges to each technology.

**Key Findings and Conclusion:**

Noteworthily, hydrolysis of dairy proteins liberate wide-range of peptides that possess remarkable biological functions to maintain human health. Novel technologies in the dairy industry such as ultrasound-assisted processing (UAP), microwave-assisted processing (MAP), and high pressure processing (HPP) are innovative and environmentally friendly. Generally, novel technologies are less effectual compared to conventional methods, therefore used in combination with fermentation and enzymatic hydrolysis, and are promising pretreatments to modify peptides’ profile, improve the yields, and high liberation of bioactive peptides as compared to conventional technologies. UAP is an innovative and most efficient technology as its mechanical effects and cavitation change the protein conformation, increase the biological activities of enzymes, and enhance enzymatic hydrolysis reaction rate.

## Highlights

- Novel technologies are innovative, environmentally friendly, and promising pretreatments.
- Mechanisms and applications of novel technologies as pretreatments have been discussed.
- Novel technologies coupled with conventional methods are energy efficient and result high extraction yield and rate have been reviewed.
- Potential bioactivity and functions of dairy proteins have been discussed.
- Ultrasound assisted processing showed most efficient applications in dairy industry have been outlined.

## Introduction

Bioactive peptides are specific peptide motifs of 2–20 amino acids embedded in parent proteins that possess the ability to alter or influence metabolic activities in the human body because of their particular fragments in proteins (1, 2). Bioactive peptides offer several biological functionalities such as free radicals inhibition, thrombosis inhibition, and immunity improvement (3). There are two main methods of bioactive peptides production by microbial fermentation and enzymatic hydrolysis of proteins. A wide range of bioactive peptides can be produced using different cleavage specificities of the proteolytic enzymes (4). Usually, bioactive peptides consist of less than 20 amino acids and 10 kDa molecular weight. Their functionalities also depend upon the sequence of amino acids, their compositions, and molecular weights (5). Milk proteins contain several peptides that exert strong biological properties and are widely studied as a source of bioactive peptides (6, 7). Many studies have reported the availability of bioactive peptides in milk, fermented dairy products, and various types of cheese (8–10). Milk derived bioactive peptides are associated with many health beneficial effects, including immunomodulation, antithrombotic activity, antihypertension, antimicrobial activity, and opiate activity (11, 12).

**FIGURE 1:**
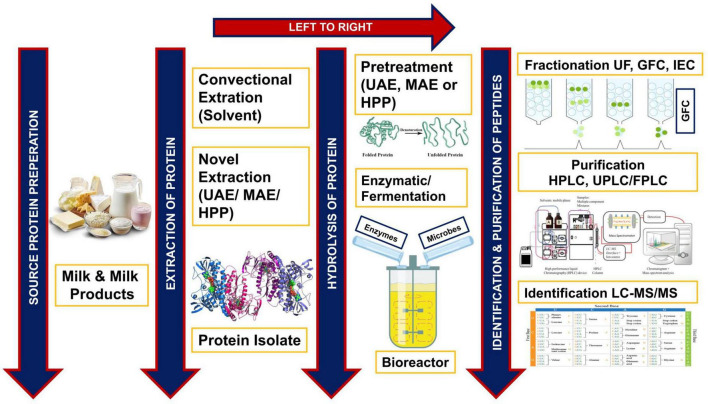
Schematic diagram of the production of bioactive peptides from dairy products.

The bioactive peptides are separated, identified, and purified by employing high-performance liquid chromatography (HPLC) (5). However, characterization of peptides is carried out by the protein hydrolyzed fractionation method (13) and functional properties of peptides are assessed by the amino acid composition of the bioactive peptide (14). For fractionation, the ultrafiltration membrane system is the preliminary step to separate the required molecular weight fractions from hydrolyzates (15). Figure 1 illustrates and summarizes the process of bioactive peptides production from dairy proteins, including source preparation, extraction, and hydrolysis of protein (denaturation), fractionation of desired peptides through gel-filtration chromatography (GFC), their purification by HPLC, and identification through liquid chromatography-mass spectrometry (LC-MS/MS).

**FIGURE 2:**
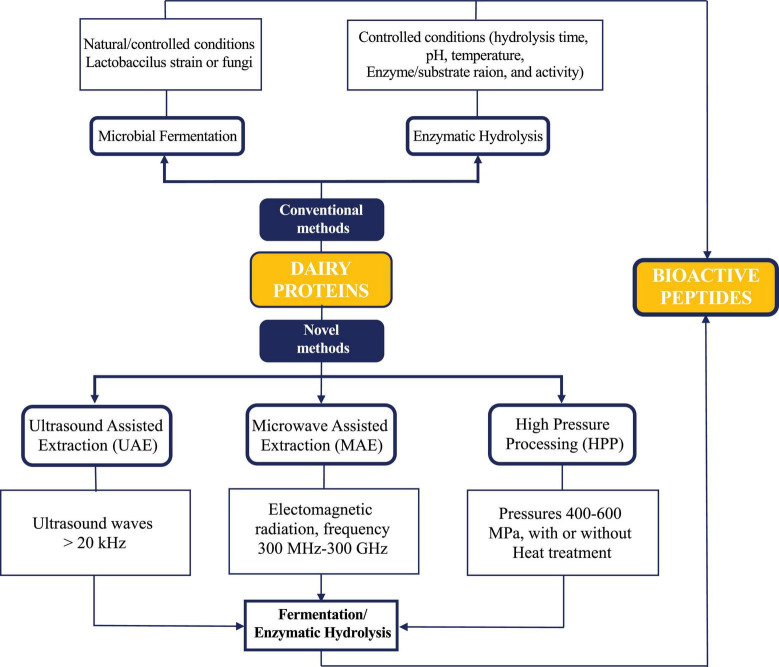
Novel and conventional methods of bioactive peptides production from dairy proteins.

The dairy industry relies on microbial fermentation and enzymatic hydrolysis to produce bioactive peptides, which alone give low yields of peptides. Various novel technologies are evolving, coupled with conventional methods to generate high yields of bioactive peptides from dairy proteins quickly and at a low cost. Figure 2 illustrates and summarizes the conventional and green novel technologies employed in the dairy industry to produce bioactive peptides. Ultrasound, microwave (16) and high-pressure processing (17) are the efficient, novel, green technologies, but these are emerging technologies with attention to dairy industry, and their promising effects have been entirely understood when employed as pretreatments. Ultrasound waves break, weaken, or clean the electrostatic and hydrophobic interactions of milk proteins through shear forces and cavitation and bring conformational changes in proteins (18, 19). Microwave heating has many benefits like easy operation, less processing, and high efficient energy, making it suitable in continuous food processing (20). High-pressure processing (HPP) is a potential technique used as a pre-treatment method to release bioactive peptides by enhancing the enzymatic digestibility of proteins due to conformational changes in proteins that influence their functional properties boosting their digestibility. It has also been applied to milk and milk products (21, 22).

This review emphasizes the production of peptides from dairy proteins and discusses the potential use of novel technologies in context to conventional methods of bioactive peptides production from dairy proteins, including the mechanisms and their respective examples of use, advantages, limitations, and challenges to each technology.

## Milk and Fermented Dairy Products: Source of Bioactive Peptides

**FIGURE 3:**
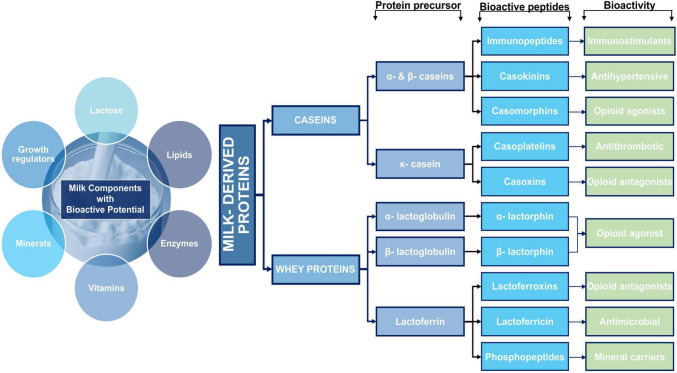
Major milk components with bioactive potential.

Milk and dairy products comprise various essential nutrients such as bioactive agents (antioxidants), minerals, omega-3 fatty acids, linoleic acid, oleic acid, and vitamins, making them nutritious foodstuff (23). Oxidative stress and damage to the body can be prevented by consuming antioxidant-rich foods (24). Milk and its products are a well-known source of antioxidants as they contain: significant amounts of daidzein polyphenolic metabolites, antioxidative enzymes, i.e., glutathione peroxidase, catalase, superoxide dismutase, and sulfur-containing amino acids, i.e., carotenoids, vitamins A and E, cysteine, and methionine (25). Generally, bovine milk protein is comprised of lactoferrin, caseins, immunoglobulins, beta-lactoglobulin (β-LG), alpha-lactalbumin (α-LA), fractions of protease-peptide, and some whey proteins (transferrin and serum albumin) as main fractions (26). Figure 3 shows the major bioactive components of milk with biological properties.

Milk contains various useful molecules encompassing bioactive peptides (27, 28). Dietary proteins contain bioactive peptides in them, which are naturally found inactive in parent protein sequences and liberated only during food processing or gastrointestinal digestion. Peptides work as regulatory compounds with hormone-like activity after liberation. In dairy, milk proteins are the potent source of bioactive peptides which exert various biological functions, i.e., antioxidant, antimicrobial, anticancer, and anti-hypertensive factors (29, 30). As cited in Table 1, many researchers have assessed the biological activities of bioactive peptides from various milk sources, including camel and bovine casein hydrolyzates (31), buffalo casein (32), camel whey protein hydrolyzate (33), camel milk lactoferrin (34), goat milk (35), yak milk (36), goat milk (37), buffalo milk (38), skim milk (6) camel milk (39), whey protein hydrolyzate (40), UHT treated milk (41), and milk and dairy products (42) by using various microorganisms and microbial enzymes for proteolysis. The bioactive peptides are liberated during gastrointestinal digestion (*in vivo*), milk products’ manufacturing, and proteolysis (*in vitro*).

**TABLE 1:** Bioactive peptides released from milk proteins by various microorganisms and microbial enzymes.

References	Microorganisms/Microbial enzyme	Protein fragment	Amino acid sequence	Bioactivity
Mudgil et al. (31)	Alcalase and pronase E	NR	FLWPEYGAL, LPTGWLM, MFE, GPAHCLL HLPGRG, QNVLPLH, PLMLP	Anti-diabetic [inhibition of α-amylase (AA), α-glucosidase (AG), and dipeptidyl peptidase IV (DPP-IV)]
Shanmugam et al. (32)	Pepsin, trypsin, chymotrypsin and their combination	α S1 Casein	HIQKEDVPSER, EDVPSER	ACE inhibitory
		α S2 Casein	EQLSTSEENSK, NPWDQVK, YQGPIVLNPWDQVK, RNAVPITPTL, NAVPITPTLNR, NAVPITPTL	
		β Casein	IHPFAQTQSL, YQEPVLGPVR, VLPVPQK, YPVEPFTESQSL	
		κ Casein	YIPIQYVLSR, YPSYGLNYYQQKPVAL, HPHPHLSF	
Baba et al. (33)	Pepsin	NR	PAGNFLMNGLMHR, PAVACCLPPLPCHM, MLPLMLPFTMGY, PAGNFLPPVAAAPVM	α-amylase and α-glucosidase inhibitory
Khajeh et al. (34)	*S. aureus*, *P. aeruginosa*, and *A. baumannii*	NR	IAGKCGLVPVL, AASKKSVRW, CTTSPAESSKCAQ, ECIQAISTEKADAVT, LRPIAAEV, GTENNPQTH, KSCHTGL, …, RRCSTSP	Antimicrobial
Panchal et al. (35)	*Lactobacillus fermentum* (M2)	NR	SCQDQPTTLAR, TIDMESTEVFTKK, YIQKEDVPSER	Antioxidative
Liu et al. (36)	Alcalase Trypsin	Yak-CN	RELEEL, GKEKVNEL, LPVPQ, HPHPHL, VLPVP, VPYPQ	Antioxidative
Parmar et al. (37)	*L. fermentum* (M5) (KU366365) *L. paracasei* (M16) (KU366368) *L. rhamnosus* (NK2) (KR080695) *L. casei* (NK9) (KR732325) *L. fermentum* TDS030603 (MTCC 25067)	CASA1_CAPHI Alpha-S1-casein OS	LARPKHPINHRGLSPE, ENSGKTTMPLW	ACE inhibitory Antihypertensive
		CASA2_ CAPHI Alpha-S2-casein OS	TEEEKNRLNFLKKISQY, PEEIKITVDDKHYQKALNEI	
Zhao et al. (38)	*Dregea sinensis* Hemsl. protease.	α_S1_- CN (f106–117)	YLGYLEQLLRLK	Antimicrobial
Guzmán-Rodríguez et al. (6)	*Lactobacillus casei* SHIROTA	β-CN κ-CN	NR	Iron binding Antithrombotic
Wali et al. (39)	Trypsin Pepsin Alcalase Papain	NR	RLDGQGRPRVWLGR TPDNIDIWLGGIAEPQVKR VAYSDDGENWTEYRDQGAVEGK	Antioxidative
Jiang et al. (40)	Trypsin	α-La (113–117), (115–123), (109–122), (94–108), (99–114), (63–79), (80–98)	KILDK, LDQWLCEKL, ALCSEKLDQWLCEK, KILDKVGINYWLAHK, VGINYWLAHKALCSEK, NDQDPHSSNICNISCDK, FLDDDLTDDIMCVKKILLDK	Antioxidative
		β-Lg (149–162), (61–75), (125–141), (102–124)	LSFNPTQLEEQCHI WENGECAQKKIIAEK TPEVDDEALEKFDKALK YLLFCMENSAEPEQSLACQCLVR	
Özturk and Akin (60)	*Lactobacillus casei* Shirota *Lactobacillus johnsonii* LA1	α-La β-Lg	NR	Antithrombotic
Elkhtab et al. (41)	Lactic acid bacteria strains	κ-CN	LVESPPELNTVQ, VLESPPELN, RSYPSYGIN	ACE inhibitory Antihypertensive
		β-CN	DQIHPFAQTK	
	Kombucha culture	α_S1_-CN	AVPQEVLNENLLR, FVAPEPFVFGKEK	
		α_S2_-CN	KFKGFVEPFPAVE, VAPFPEVFGK	
		β-CN	LVYPFPGPLH, LVYPFPGLPAAPVLPQ	
Capriotti et al. (42)	*Lactobacillus helveticus*	β-CN (205–209)	FPIIV	ACE inhibitory

*NR, not reported; α_S1_-CN, alpha-S1-casein; α-La, alpha lactalbumin; κ-CN, kappa-casein; β-CN, beta-casein; β-lg, beta lactoglobulin; CASA1_CAPHI OS, Capra hircus alpha-S1-casein; CASA2_ CAPHI Alpha-S2-casein OS, Capra hircus alpha-S2-casein.*

Reportedly, fermented milk products contain phosphor-peptides, ACE-inhibitory peptides, and casomorphins (43). Bovine α-lactalbumin and β- casein have shown bioactive peptide sequences like LDQW, INYW, and NSLP, FP, HQP, respectively (44, 45). Another *in vitro* study revealed two antioxidative peptide sequences KVLPVPEK and AVPYPQR, by following milk casein hydrolysis (46). However, digestion and fermentation of goat milk can also release antioxidative peptide sequences like EALEKFDK and EALEKFDK (47, 48).

Cheese is a widely used fermented milk product. Many studies have reported that cheese is a vital source of wide-range of biologically active substances such as proteins and all essential amino acids (except cysteine and methionine), minerals, vitamins, and short-chain fatty acids (49, 50). Cheese contains bioactive compounds with biological activities such as peptides, conjugated linoleic acid (CLA), exopolysaccharides, γ-aminobutyric acid (GABA), vitamins, and organic acids, and fatty acids. According to *in vitro* and *in vivo* studies, these bioactive compounds may have antiproliferative, antimicrobial, and antioxidant activities and inhibit ACE (angiotensin-converting enzyme) (51, 52). As shown in Table 2, many bioactive peptides have been identified in different fermented dairy products, such as Iranian ultrafiltered white cheese (53), fermented milk (54), cultured dairy product (55), Hard cow milk cheese (56), fermented casein (57), Prato cheese (58), fermented whey proteins (59), commercial fermented milk (60), goat milk Tulum cheese and cow milk Tulum cheese (61), cow and buffalo cheddar cheeses (62), fermented milk (Lassi) (63), yogurt (64), symbiotic yogurt (65), and curd and whey (66).

**TABLE 2:** Bioactive peptides identified in fermented dairy products.

References	Product	Protein fragment	Amino acid sequence	Bioactivity
Yousefi et al. (53)	Iranian ultrafiltered white cheese	α_S1_-CN (1–6) α_S1_-CN (102–108)	RPKHPI, KKYNVPQ	ACE inhibitory
		β-CN (f205–209), (f126–133), (f114–121), (f57–68), (f193–209)	FPIIV, FPKYPVEP, YPVEPFTE, SLVYPFPGPIHN, YQEPVLGPVRGPFPIIV	
Kim et al. (54)	Fermented milk	NR	ATISAG	Lipase Inhibitory
Mullaiselvan et al. (55)	Cultured dairy product	α_s1_-CN α_s2_-CN β-CN	NR	Casein phosphopeptide Immunomodulatory
Timón et al. (56)	Hard cow milk cheese	α_s1_-CN β-CN	EIVPN, DKIHPF, VAPFPQ	Antioxidative
Fan et al. (57)	Fermented casein	α-La (113–117), (115–123), (109–122), (94–108), (99–114), (63–79), (80–98)	KILDK, LDQWLCEKL, ALCSEKLDQWLCEK, KILDKVGINYWLAHK, VGINYWLAHKALCSEK, NDQDPHSSNICNISCDK, FLDDDLTDDIMCVKKILLDK	Antioxidative
		β-Lg (149–162), (61–75), (125–141), (102–124)	LSFNPTQLEEQCHI WENGECAQKKIIAEK TPEVDDEALEKFDKALK YLLFCMENSAEPEQSLACQCLVR	
Baptista et al. (58)	Prato cheese	β-CN (f194–209)	NR	ACE inhibitory
Daliri et al. (59)	Fermented whey proteins	α_S1_-CN (f10–23), (f10–22), (f1–23), (f14–23), (f10–21), (f24–34), (f24–38), (f80–98)	GLPQEVLNENLLRF, GLPQEVLNENLLR, RPKHPIKHQGLPQEVLNENLLRF, EVLNENLLRF, GLPQEVLNENLL, FVAPFPEVFGK, VAPFPEVFGK, FVAPFPEVFGKEKVNEL, HIQKEDVPSERYLGYLEQL	ACE inhibitory Antihypertensive
		β-CN (f1–27), (f1–25), (f1–22), (f1–24), (f192–209), (f193–209), (f193–208), (f194–209), (f195–209), (f83–95)	RELEELNVPGEIVESL, RELEELNVPGEIVE, RELEELNVPGE, RELEELNVPGEIV, LYQEPVLGPVRGPFPIIV, YQEPVLGPVRGPFPIIV, YQEPVLGPVRGPFPII, QEPVLGPVRGPFPIIV, EPVLGPVRGPFPIIV, VVPPFLQPEVMGV	
		κ-CN (f161–169), (f155–169), (f149–169), (f151–169), (f159–169), (f152–169), (f150–169), (f157–169), (f151–169), (f151–165), (f151–163), (f149–162), (f149–163), (f151–162), (f116–141), (f109–151), (f106–149)	TVQVTSTAV, SPPEINTVQVTSTAV, SPEVIESPPEINTVQVTSTAV, EVIESPPEINTVQVTSTAV, INTVQVTSTAV, VIESPPEINTVQVTSTAV, PEVIESPPEINTVQVTSTAV, PEINTVQVTSTAV, EVIESPPEINTVQVTSTAV, EVIESPPEINTVQVT, EVIESPPEINTVQ, SPEVIESPPEIN, SPEVIESPPEINTVQ, EVIESPPEIN, MAIPPKKNQDKTEIPTINTIASGEPT, PPKKNQDKTEIPTINTIASGEPT-STPTTEAVESTVATLEDSPE, MAIPPKKNQDKTEIPTINTIASGE-PTSTPTTEAVESTVATLED	
		β-lg (f130–149), (f130–146), (f130–145), (f1–11), (f153–162), (f147–156), (f1–11), (f1–12), (f153–162), (f147–156), (f1–10)	DEALEKFDKALKALPMHIRL, DEALEKFDKALKALPMH, DEALEKFDKALKALPM, LIVTQTMKGLD, PTQLEEQCHI, IRLSFNPTQL, LIVTQTMKGL, LIVTQTMKGLD, PTQLEEQCH, IRLSFNPTQL, LIVTQTMKGL,	
		Lactophorin (PP3) (f1–18), (f1–17), (f57–67), (f54–67)	ILNKPEDETHLEAQPTDA, ILNKPEDETHLEAQPTD, QPQSQNPKLPL, SSRQPQSQNPKLPL	
		PIGR (f383–404)	PGRPTGYSGSSKALVSTLVPLA	
		UP (GP2) (f455–473)	SEGVAIDPARVLDLGPITR	
Pérez-Escalante et al. (60)	Commercial fermented milks	α-La β-Lg	NR	Antithrombotic
Özturk et al. (61)	Goat milk Tulum cheese Cow milk Tulum cheese	NR	NR	Antimicrobial
Rafiq et al. (62)	Cow Cheddar cheese Buffalo cheddar cheese	α-CN β-CN	NR	Antihypertensive Antithrombotic
Padghan et al. (63)	Fermented Milks (Lassi)	β-CN (f47–56), (f47–57), (f199–209), (f176–182), (f176–183), (f176–184), (f1–7), (f57–68), (f166–175), (f195–206), (f195–207), (f195–209), (f94–106), (f169–176)	NR	ACE inhibitory Immunomodulatory Antioxidative Opioid Cytomodulatory
Jin et al. (64)	Yogurt	β-CN (f1–27), (f1–25), (f1–22), (f1–24), (f192–209), (f193–209), (f193–208), (f194–209), (f195–209)	RELEELNVPGEIVESL, RELEELNVPGEIVE, RELEELNVPGE, RELEELNVPGEIV, LYQEPVLGPVRGPFPIIV, YQEPVLGPVRGPFPIIV, YQEPVLGPVRGPFPII, QEPVLGPVRGPFPIIV, EPVLGPVRGPFPIIV	ACE inhibitory Antihypertensive
Sah et al. (65)	Symbiotic yoghurt	β-CN	YQEPVLGPVRGPFPIIV, SLPQNIPPLTQTPVVVPPF	Antiproliferative Antioxidative
Dabarera et al. (66)	Curd Whey	Di and tripeptides Penta-octapeptides	Closely similar to YGGFM YGGFL, IPI	ACE inhibitory Antihypertensive

*NR, not reported; α_S1_-CN, alpha-S1-casein; α_S2_-CN, alpha-S2-casein; α-CN, alpha-casein; α-La, alpha lactalbumin; κ-CN, kappa-casein; β-lg, beta lactoglobulin; β-CN, beta-casein; UP (GP2), uncharacterized protein GP2; PIGR, polymeric immunoglobulin receptor.*

## Conventional Methods of Production

In the dairy industry, the conventional methods for producing bioactive peptides are microbial fermentation and enzymatic hydrolysis, summarized in Table 1.

### Microbial Fermentation

Fermentation is a primeval preservation method that utilizes lactic acid bacteria proteolytic systems as an efficient approach to produce bioactive peptides from food. Generally, lactic acid bacteria fermentation is carried out both naturally and under controlled conditions, which improve technological and nutritional properties of food and ultimately develops texture and flavor in them (67, 68). Usually, the milk fermentation is carried out by Lactobacillus strains; till now, most known bioactive peptides have been isolated through milk cultures (27). Because different milk sources (cow, buffalo, goat, yak, camel, or mare) have distinctive proteins, different bioactive peptides are produced on hydrolysis of casein and whey proteins usage of the same Lactobacillus strain (69). Lactic acid bacteria fulfill their need for essential and growth-promoting amino acids from milk proteins as a primary source (70). Microbial fermentation is an efficient and economical method to produce peptides (71) which is extensively employed to functionalize milk products and byproducts in the dairy industry (72, 73). During the manufacturing of fermented dairy products starter and non-starter, bacteria can produce bioactive peptides because of the high proteolytic activities exerted by dairy starter cultures (74). Ueno et al. (75) and Phelan et al. (76) utilized *L. helveticus* CM4 to produce an endopeptidase that possessed the ability to produce antihypertensive peptides by using synthetic pro-peptides as a substrate. Also, lactic acid bacteria have helped achieve multifunctional bioactive peptides (77, 78). Zanutto-Elgui et al. (79) have reported the production of bioactive peptides having antioxidant and antimicrobial activity from goat and bovine milk by using the proteolytic activity of *Aspergillus flavipes* and *Aspergillus oryzae* enzymes.

Microbial fermentation is comparatively economical than the enzymatic method for bioactive peptides production. Microbial fermentation method applications have some industrial limitations as they yield low peptide production and lack specificness of peptide generation (69).

### Enzymatic Hydrolysis

Enzymatic hydrolysis is a reliable, efficient, and the principal method to hydrolyze whole proteins for the production of bioactive peptides under the mild condition of enzyme activity, substrate concentration, hydrolysis time, temperature, and pH. These peptides exert anti-inflammatory, opioid, immunomodulatory, anticancer, antioxidant, antithrombotic, and antihypertensive activities (67, 80). The results of the efficiency of enzymatic hydrolysis mostly depend on two factors: the primary structure of parent protein (substrate) and specificity of the enzyme applied (81). Animal, plant (neutrase, thermolysin, ficin, pronase, flavourzyme, and papain), microbial, and digestive (chymotrypsin, trypsin, and pepsin) origin enzymes have been used to hydrolyze the large sequence peptides into small sequence peptides having 500–1,800 kDa molecular weights and 2–20 amino acid units (82, 83). *In vitro* studies have shown that the parent milk proteins undergo hydrolysis with pancreatic proteinases (mostly trypsin) and liberate most known biological peptides. Though, endoprotease combinations (proline-specific endopeptidase, carboxypeptidase, elastase, pancreatin, thermolysin, pepsin, and chymotrypsin) are also reported to produce bioactive peptides (84, 85). Furthermore, intact protein molecules can be hydrolyzed by combining enzymes like Thermolysin, Trypsin, Pancreatin™, Chymosin, and Alcalae™ to produce bioactive peptides (76). Combinations of carboxypeptidase, elastase, chymotrypsin, trypsin, and pepsin have been used to liberate various α-lactalbumin and β-lactoglobulin corresponding fractions and ACE-inhibitory peptides having different IC_50_ values (84–86). Liu et al. (36) isolated the antioxidative peptides (RELEEL) from the yak casein hydrolyzate using the combination of alcalase and trypsin digestion. Abdel-Hamid et al. (87) subjected buffalo skimmed milk to hydrolysis using papain, pepsin, trypsin, and isolated known and novel ACE inhibitory antioxidative peptides. Wali et al. (39) used a combination of trypsin, pepsin, alcalase, and papain to hydrolyze the Bactrian camel milk and isolated three novel antioxidant peptides.

Enzymatic hydrolysis has certain shortcomings, such as higher cost to produce pure bioactive peptides, casein coagulation on heating, and bitterness, therefore, choice of enzymatic hydrolysis conditions must be taken into account before application (88, 89).

## Novel Processing Technologies

The novel processing technologies such as ultrasound-assisted processing (UAP), microwave-assisted processing (MAP), high-pressure processing (HPP), pulsed electric field processing (PEF), subcritical water processing (SWP), and ohmic heating relies on physical processes to improve the degree of hydrolysis during bioactive peptides production (90, 91). However, following applications of UAP, MAP, and HPP as pretreatments have been found in the dairy industry to prepare bioactive peptides.

### Ultrasound-Assisted Processing

Ultrasound-assisted processing is a novel, eco-friendly, and non-thermal physical technology that involves >20 kHz frequency of sound waves to produce peptides (91, 92). In ultrasound treatment, acoustic cavitation, acoustic streaming, and mechanical vibrations are produced on the passage of ultrasound waves through a medium. Acoustic streaming can allow and improve the transfer of mass through a medium. The mechanical vibrations can change solid particle size and structure (93). In a liquid medium, ultrasound treatment follows the cavitation process in which pre-existing micro-bubbles expand and contract. However, during these oscillations, bubbles keep growing until they reach their resonance size range and then collapse violently in case of transient/inertial cavitation (94, 95). In transient cavitation, physical shearing, high-pressure and extreme localized temperatures (2,000–5,000 K) are produced on collapsing of increased sized bubbles (within few acoustic cycles) into fragments at low ultrasound frequency. However, stable cavitation results in relatively mild streaming effects on collapsing of the little increased bubbles (over a large number of acoustic cycles) at higher frequencies. Cavitation also owns the ability to induce chemical changes along with physical effects. When cavitation is applied to an aqueous medium, a highly reactive radical is formed inside the bubble (on reaction of gas molecules and water vapor reaction) due to the availability of generated localized high temperature. The ultrasound cavitation chemical effects are visible at 300–500 kHz frequencies and physical effects are visible at 20 kHz frequency (94). Protein structures undergo conformational changes by ultrasound processing, such as acoustic cavitation, forces of chemical and physical effects (96, 97).

As presented in Table 3, recently UAP has been employed as pretreatment for various milk proteins hydrolysis, including whey proteins (98), caprine milk protein (99), fresh milk (100), cheddar cheese (101), whey protein isolate (102), whey protein (103), and milk protein concentrate (104, 105). UAP in combination with enzymatic hydrolysis has been employed for various proteins, i.e., eggshell membrane (106), egg white (107), and isolated oat protein (108). Ulug et al. (91) reported that the application of UAP is carried out in combination with enzymatic hydrolysis, to increase the production of bioactive peptide, as UAP alone cannot break the peptidic bond. Ultrasound pretreatment enhance the enzymes accessability into the peptide bonds of foods that results in the increased release of bioactive peptides. Basically, ultrasound processing generates the acoustic forces that increase the available surface area for enzyme protein interactions by reducing the size of the fat globules that get covered with whey proteins and casein micelles, ultimately, increases the access of proteolytic enzymes to the proteins (109, 110). Wu et al. (111) in their study on the thermodynamic properties of whey protein hydrolyzed by alcalase with ultrasonic pretreatment reported that the hydrolyzates showed significantly increased ACE inhibitory and immunomodulatory activities when the whey protein enzymatic hydrolysis was assisted by the ultrasound. Sonication pretreatment induces the whey protein unfolding, increased free sulfhydryl content, and conformational changes with increased β-sheets and β-turns formation (111). Similar study exhibited that ultrasound-assisted pretreatment combined with low purity enzymes show the increased hydrolysis rate that may be due to changes in free sulfhydryl clusters and disulfide bond (112), hydrophobic protein content, and surface hydrophobicity (113). Lorenzetti et al. (102) reported that ultrasound pretreatment before hydrolysis of whey protein isolate could help to develop the economic ingredients for the dairy industry.

**TABLE 3:** Applications of ultrasound- assisted processing for the production of bioactive peptides.

References	Protein source	Equipment	Type of treatment	Treatment conditions	Peptides/hydrolyzate size	Major findings
Abadía-García et al. (98)	Whey proteins	Probe ultrasound homogenizer	The high intensity ultrasound (HIUS) pretreatment before enzymatic hydrolysis (bromelain)	The ultrasonic pretreatment at 500 W, 20 kHz, 25 and 50% amplitude, 10 min	Higher concentration of peptides with a molecular weight below 5 kDa was found when ultrasound pretreatment was applied.	In comparison to control, both HIUS pretreatments resulted reduced the IC50 value in hydrolyzates, small size fractions (1 and 3 kDa) showed highest ACE inhibition activity, and significant changes were observed in structure of whey protein.
Koirala et al. (99)	Caprine milk protein	Probe sonicator	The ultrasonic pretreatment before enzymatic hydrolysis (pepsin and neutral protease)	200 W power, 24 kHz frequency and a fixed cycle of 0.5	Ultrasonic pre-treated caprine milk proteins had a higher degree of hydrolysis with neutral protease at 360 min and with pepsin at 300 min. The molecular weight of peptides after sonication was not measured.	The ultrasonication pretreatment increased the soluble protein concentration in caprine milk, enhanced peptides and protein hydrolyzates production, and accelerated unfolding of complex insoluble protein structure into a simpler soluble matrix, and increased bioactive antioxidant and ACE-inhibitory activities.
Cui et al. (100)	Milk protein	Multi-mode ultrasonic	The ultrasonic pretreatment before enzymatic hydrolysis (neutral protease)	Single frequency 28 kHz, various times ranging 10–60 min, different levels of ultrasound density between 10 and 50 W/L at initial temperature 30°C.	Ultrasonic pre-treated milk proteins had a higher degree of hydrolysis than the non-ultrasound samples. The molecular weight of peptides after sonication was not measured.	Compared with control and non-ultrasonic samples, the ultrasonic pretreatment showed significantly increased ACE inhibitory activity of milk protein (28 kHz, 20 W, and 40 min). Also, secondary structure studies showed reduced content of α-helix and β-corner, increased content of β-folding, and random coil in ultrasonic treated milk proteins. And, increased surface hydrophobicity and the content of free sulfhydryl, reduced content of disulfide bond in ultrasonic pretreated milk protein.
Munir et al. (101)	Cheddar cheese	Probe sonicator	The ultrasonic pretreatment of milk before cheddar cheese manufacturing and compared with control and other processing techniques.	80% amplitude 20 kHz frequency at <40°C. Applied in two levels: US-1 (21 J/g calorimetric power) & US-2 (41 J/g)	The molecular weight of peptides after sonication was not measured.	In comparison to control, both levels of ultrasonic treatments increased the proteolysis process of cheese as well as fat content, ACE-inhibition activity, total phenolics, total flavonoids, antioxidant and DPPH scavenging activities of the cheddar cheese during ripening.
Lorenzetti et al. (102)	Whey protein isolate	Ultrasonic tip sonicator	The ultrasonic pretreatment before enzymatic hydrolysis (low purity enzymes: pepsin and papain)	20 kHz frequency, pepsin (4 min at 400 W), papain (2 min at 300 W)	The highest degree of hydrolysis reported from pepsin. The molecular weight of peptides after sonication was not measured.	The ultrasonic pretreatment reduced the 6 h in the process. The highest degree of hydrolysis occurred with the use of pepsin (10 h, 37°C, and pH 2.5). After partial enzymatic hydrolysis and ultrasound pretreatment a higher proportion of low molar mass peptides were observed at 1,000–2,000 g.mol^–1^.
Abadía-García et al. (103)	Whey protein	Ultrasound homogenizer	The ultrasonic pretreatment before enzymatic hydrolysis (vegetable proteases)	20 kHz frequency, 750 W nominal power, amplitude between 30 and 60%.	The molecular weight of peptides after sonication was not measured.	The results showed that ultrasound density exerted a significant effect on proteolysis increased the ACE inhibition by 13% and a 95% reduction of hydrolysis time in bromelain hydrolyzates. Also, changes in denaturation enthalpy (ΔH), reduction of reactive thiol groups and changes in secondary structure suggest protein rearrangements and aggregate formation.
Uluko et al. (105)	Milk protein concentrate (MPC)	Cell disruptor	The ultrasonic pretreatment before enzymatic hydrolysis with digestive enzymes (pepsin and trypsin) and compared with thermal and microwave pre-treatments.	Different combination of pretreatments were set. The ultrasonic pretreatment at 90°C, US at 800 W and 20 kHz for 10 min. Samples were jacketed with ice during treatment. Control received no pretreatment.	The molecular weight of peptides after sonication was not measured.	Compared with the control and other treatments, US pretreated samples showed the highest radical scavenging activity (EC_50_ = 0.283 mg mL^–1^) and had the highest number of hydrophobic peptides.
Uluko et al. (104)	Milk protein concentrate (MPC)	Ultrasonic homogenizer	The ultrasonic pretreatment before enzymatic hydrolysis (neutrase)	Different combinations of independent variables were set (pre-treatment time, hydrolysis time, and enzyme/substrate (E/S) ratio)	The optimal ultrasonic pre- treatment significantly increased the degree of hydrolysis.	According to response surface analysis, the highest ACE inhibitory activity (IC_50_ = 0.044 mg mL^–1^) could be achieved by 4.11 min, 2.32 h and 2.33% for ultrasound pretreatment time, hydrolysis time and E/S ratio, respectively. Also, the ultrasound pretreatment has a significant effect on ACE inhibition of enzyme hydrolyzates from MPC during enzymatic hydrolysis with digestive enzymes.

UAP application is beneficial to reduce the disadvantages resulting from hydrolysis by conventional enzymes, i.e., long-time hydrolysis and low conversion rate (114). Generally, UAP equipment requires fewer installations, low maintenance, around 85% energy efficiency, and cost between €10,000 and 200,000 (115). Undeniably, UAP is one of the novel and most preferable techniques for producing bioactive peptides due to numerous advantages such as faster start-up, extraction selectivity, high process control, reduced temperature and time, and faster mass and energy transfer (116).

### Microwave-Assisted Processing

Microwaves encompass electromagnetic radiation of 300 MHz–300 GHz range (117). Microwave energy follows molecular interactions (ionic conduction and dipolar rotation mechanisms) as a medium transportation mode. On applying electromagnetic field, charged colloidal molecules migrate and flow through a stationary medium in ionic conduction and led to resistance in the solution, which produces thermal energy. On the other hand, dipole rearrangement occurs on electromagnetic fields in dipolar rotation (118).

Microwave treatment is carried out in the food processing ovens in which an alternating electric field is used to generate the microwaves having 2.45 GHz frequency and <1 cm wavelength typically. These microwaves do not cause breakage of covalent bonds because of their non-ionizing radiation nature (119, 120) but, these can either induce thermal or non-thermal changes in the milk. Microwaves generate heat by friction that results from the oscillation of molecules as dipoles of water try to align their arrangements under the influence of microwave field. So, thermal effects are resulted from the generation of localized heat due to friction of molecules, on the other hand, non-thermal effects (accelerated protein unfolding rate) alone arise from the rearrangement of molecules in milk (120).

Microwave-assisted processing has been employed for various milk proteins hydrolysis including cheddar cheese (101), bovine whey proteins (121), milk protein concentrate (105), bovine serum albumin (122), and bovine whey protein concentrate (123) as cited in Table 4. MAP is one of the most preferred alternative technologies to conventional heat processing methods as it enhances functional properties, extends shelf life, and improves microbial safety of food products (124, 125). In their study, Izquierdo and coworkers found that MAP could make proteins specific sites potentially available to proteolytic enzymes by continuous protein molecules unfolding and rearrangement (123). In a study, the surface plasmon resonance sensing method was used to investigate the unfolding of protein by employing MAP at 2.45 GHz. The results showed that at the same temperature, MAP heating has a higher impact on the unfolding and denaturation of a bovine crystalline than conventional heating (126).

**TABLE 4:** Applications of microwave-assisted processing for the production of bioactive peptides.

References	Protein source	Equipment	Type of treatment	Treatment conditions	Peptides/hydrolyzate size	Major findings
Munir et al. (101)	Cheddar cheese	Microwave oven	The microwave pretreatment of milk before cheddar cheese manufacturing and compared with control and other processing techniques.	Temperature <40°C, specific energy 86.5 J/g	The molecular weight of peptides after microwave treatment was not measured.	In comparison to control, MA showed increased antioxidant activity and ACE-inhibitory potential of cheese. However, ultrasound was the most effective pre-treatment to improve the antioxidant capacity of cheddar cheese during ripening.
El Mecherfi et al. (121)	Bovine whey proteins	Microwave device consisted of a solid-state microwave generator	Microwave pre-treatment followed by proteolysis (pepsin), and compared with conventional heating.	Different microwave temperatures conditions at 37, 50, 65, and 70°C for 30 min and microwave power was not reported	The highest degree of hydrolysis reported from pepsin compared to conventional heating. Whey proteins showed two major bands with molecular weights: 18 kDa bovine beta-lactoglobulin and 14 kDa alpha-lactalbumin.	The microwave heating process in concomitance with enzymatic proteolysis improved the susceptibility of resistant proteins (BLG) to pepsinolysis. Also, hydrolyzed whey protein hydrolyzates were obtained by MA only at 65°C and in a shorter time compared with the conventional thermal treatment.
Uluko et al. (105)	Milk protein concentrate (MPC)	Microwave	Microwave pre-treatment followed by enzymatic hydrolysis with digestive enzymes (pepsin and trypsin) and compared with thermal and ultrasound pre-treatments.	Samples were microwaved for 10 min and microwave power was not reported	The peptides have been concentrated in the filtrates of 5 kDa molecular weight	Microwave pretreated filtrates (<5 kDa) improved the radical scavenging activity compared to control; however, when microwave pretreatment was used in combination with other treatments, the samples showed lower radical scavenging activity than the control. Ultrasound was the most effective pre-treatment to improve the antioxidant capacity of milk protein concentrate.
Chen et al. (122)	Bovine serum albumin (BSA)	MAS-II Smart Microwave Digestion System	Continuous microwave-assisted protein digestion with an immobilized enzyme (trypsin)	Continuous microwave power at 100–700 W for 5–20 min for BSA digestion.	The molecular weight of the BSA- derived peptides ranged from 3 to 14 kDa (at 300, 500, and 700 W)	The bioactivity of peptides was not measured. Continuous microwave- assisted enzymatic digestion with immobilized enzyme was a fast and efficient digestion method for protein. Different levels of microwave power significantly affected the number of peptides obtained from the BSA.
Izquierdo et al. (123)	Bovine whey protein concentrate (WPC)	Oven MDS-2000	Microwave pre-treatment followed by proteolysis (pronase, chymotrypsin, papain, corolases 7089 and PN-L 100, alcalase and, neutrase)	532 W, 40 or 50°C during 5 min	The molecular weight of peptides after microwave treatment was not measured.	Microwave irradiation (MWI) treatment enhanced the enzymatic hydrolysis of bovine WPC. Pronase and Papain showed the highest proteolysis under MWI followed by Alcalase.

Microwave is the most extensively studied and world-widely popular method in both academics and food processing industry due to high heating rates which eventually lead to a clean environment of work, easy operation, low processing time, and low maintenance requirements (127, 128). In the food industry, MAP has extensive applications to extract bioactive compounds from plant materials. During extraction, MAP is used to facilitate quick heating of solvent to separate analytes and matrix. Many studies have been reported to show the efficient production of bioactive peptides from MAP as pretreatment combined with proteolytic enzymes by accelerating the rapid hydrolysis of protein into peptides and producing more coverage of sequence (129). Before proteolytic hydrolysis, cleavage sites of proteins are probably exposed by microwave radiations that cause a change in protease cleavage sites (130).

Generally, in contrast to conventional methods, MAP offers several benefits: reproducibility, reduced processing time, hydrolysis efficiency, cost-effectiveness, convenience, and simple handling, making it one of the most preferred methods (131).

### High Hydrostatic Pressure Processing

High-pressure processing (HPP) is a green, novel, and non-thermal technology that encompasses the application of 100–1000 MPa pressure, with or without treatment of heat primarily for the deactivation of pathogenic microorganisms along with molds, yeast, and vegetative bacteria, enhancing nutritional and functional properties of food products in the food industry. Depending on the food type, HPP treatment duration varies between 0 and 30 min (132, 133). Also, both treatment duration and pressure-transmitting fluid, and adiabatic heating result in a 3–9°C increase of temperature per 100 MPa (134). This technology has advantages over other technologies due to low to moderate temperature and causing the least damage to the bioactive compounds. HPP involves the combination of pressure and heat, resulting in conformational changes of protein and biological, chemical, and physical changes in food compounds (135).

High-pressure processing can be carried out in three different modes like semi-continuous, continuous, and batch. Batch HPP is an efficient and simple mode. The pressure chamber is filled with a prepacked sample and sealed, the air in the pressure chamber is replaced by pouring water, and then pressure is built until the desired point is achieved. After a particular time chamber is depressurized. Finally, processed food is taken out. On the other hand, continuous/dynamic HPP (136) involves utilizing a moving piston to push the food through a narrow gap (137). While in the case of semi-continuous HPP, the flow of liquid is introduced and contained in the same chamber at constant pressure for a specific time, after that, processed liquid food is stored in sterile tanks (138).

High-pressure processing has been employed for various milk proteins hydrolysis including whey protein concentrate (139, 140), cheddar cheese (101), bovine whey protein beta-lactoglobulin (141), whey protein isolate (142), cheddar cheese (22), beta-lactoglobulin (143), and bovine whey proteins (144) as cited in Table 5. Relatively, HPP is a well-developed technology that has many applications to milk and cheese (21, 22, 145). Munir et al. (101) reported the increased ACE Inhibitory activity HPP treated milk cheese and indicated that HPP results in efficient bioactive peptides liberation and proteolysis by imparting change in indigenous milk enzymes structures by subjecting more active sites for protein reaction (22, 146). Various studies have been reported in which the patterns of native and pressure-treated proteins have been compared. Indeed, Maynard and coworkers found that under pressurization tryptic β-LG hydrolysis generated a low concentration of intermediate hydrolysis peptides (147). On the other hand, Knudsen and coworkers reported the application of HPP at the beginning step of tryptic β-LG hydrolysis that generated an increased amount of high molecular weight peptides and hydrophobic peptides (148).

**TABLE 5:** Applications of high-pressure processing for the production of bioactive peptides.

References	Protein source	Equipment	Type of treatment	Treatment conditions	Peptides/hydrolyzate size	Major findings
Landim et al. (139)	Whey protein concentrate (WPC)	High hydrostatic pressure equipment	The HPP pretreatment of WPC	Different pressure (100, 250, and 400 MPa) and time (5, 20, and 35 min) levels for each treatment	The molecular weight of peptides after HPP treatment was not measured.	As compared to conventional hydrolysis, the HPP pretreatment increased antioxidant activity, less soluble protein hydrolyzates, and decreased allergenicity.
Paula et al. (140)	Whey protein concentrate	High hydrostatic pressure equipment	The HPP assisted hydrolysis and pretreatment of whey protein	Different pressure (100, 250, and 400 MPa) and time (5, 20, and 35 min) levels for each treatment	The molecular weight of peptides after HPP treatment was not measured.	In comparison to conventional hydrolysis, HPP assisted hydrolysis resulted in 35% protein reduction at 100 MPa after 35 min, and HPP pretreatment resulted that about 98% peptic hydrolysis of β-lactoglobulin and increased antioxidant capacity of hydrolyzates.
Munir et al. (101)	Cheddar cheese	High-pressure vessel	The HPP pretreatment of milk before cheddar cheese manufacturing and compared with control and other processing techniques.	The high-pressure processing at 400 MPa for 15 min, at temperature <40°C	The molecular weight of peptides after HPP treatment was not measured.	In comparison to control, MA and US-1, HPP showed increased antioxidant activity and ACE-inhibitory potential of cheese. However, ultrasound was the most effective pre-treatment to improve the antioxidant capacity of cheddar cheese during ripening.
Boukil et al. (141)	Bovine whey protein beta- lactoglobulin (β-LG)	Discontinuous hydrostatic pressurization unit	HHP pre-treatment followed by tryptic hydrolysis	Three different pressures at 0.1 (control), 400, and 600 MPa for 10 min at room temperature	Tryptic hydrolysis of pre-pressurized β-LG at 400 MPa generated two new peptides, (QEAKDAFLGSF and WENGECAQKK), and their relative abundance decreased at 600 MPa.	HHP pre-treatment at 400 MPa improved the generation of bioactive peptides compared to the control and 600 MPa. The relative proportions of the bioactive peptides in hydrolyzates were 38.64% at 400 MPa, higher than the control, and 600 MPa (26.7 and 20.5%, respectively).
Piccolomini et al. (142)	Whey protein isolate (WPI)	Avure High-pressure Processing System	HHP pre-treatment followed by proteolysis (pepsin, trypsin, and chymotrypsin)	Pressure levels at 550 MPa and control	High molecular weight peptides were removed with a membrane with a molecular weight cut-off 10 kDa.	Whey protein hydrolyzates with HHP treatment could reduce inflammation and oxidative stress in intestinal cells. A significant reduction of H2O2-induced IL-8 secretion was observed for the HHP treated hydrolyzates (50%) compared to the control (30%).
Voigt et al. (22)	Cheddar cheese	Equipment type not mentioned	Raw and HP-treated milk and their impact on cheddar cheese during ripening	400 or 600 MPa for 10 min at 20°C	The molecular weight of peptides after HPP treatment was not measured.	Increased proteolysis and levels of free fatty acids were found in cheese manufactured from milk HP-treated at 600 MPa
Chicón et al. (143)	β-Lactoglobulin	900 HP apparatus	HHP pre-treatment followed by proteolysis (chymotrypsin)	Pressure levels at 400 MPa	The molecular weight of peptides after HPP treatment was not measured.	Proteolysis during or after high-pressure treatment showed longer and more hydrophobic peptides than proteolysis at atmospheric pressure.
Peñas et al. (144)	Bovine whey proteins	Discontinuous high-pressure machine	HHP pre-treatment followed by proteolysis (trypsin, chymotrypsin, and pepsin)	100–300 MPa for 15 min at 37°C	The hydrolyzates obtained at 200 MPa showed two additional bands of 3 and 1.4 kDa with higher intensity than in the control. Also, the highest degree of tryptic proteolysis occurred at 200 MPa, with production of smaller peptides, in agreement with the highest degree of hydrolysis.	The high- pressure treatment enhanced the enzymatic hydrolysis of bovine whey proteins. Chymotrypsin and trypsin showed the highest proteolysis at 100 and 200 MPa followed by pepsin at 300 MPa. Bovine whey hydrolyzates obtained by pepsin and trypsin in combination with HP treatment could be used as a source of peptides in hypo- allergenic infant formulae

In the food industry, high-pressure processing is well known as a clean method compared to conventional methods as it offers numerous advantages such as homogeneous and constant pressurization at ambient temperatures, utilize less energy due to maintenance of constant pressure when reached absolute pressure, quick pressurization, and de-pressurization, reduced processing time, and it’s throughout applications irrespective of shape or size in the food system (149–151). However, the applications of this technology have certain limitations such as batch operation and costly infrastructure around 0.6–4 M US dollars accounting for 75–80% of the investment as the initial investment (152, 153). HPP has limited effects on covalent bond cleavage and production of bioactive peptides alone, therefore, it’s employed in combination with enzymatic hydrolysis to denature protein and improved access to sites of enzyme cleavage to get efficient and increased production process of bioactive peptides (91).

## Future Outlook

Numerous studies on the identification and evaluation of *in vitro* bioactivity of peptides from protein hydrolyzates of several sources of protein suggest that novel technologies should be employed to isolate novel ingredients to prepare novel functional foods. But, the application of novel technologies is an emerging field of rising significance in the dairy industry as, till now, there are minimal studies on the improvement of fermentation/enzymatic hydrolysis using UA, MA, and HPP as pretreatments to produce bioactive peptides while fermentation/enzymatic hydrolysis are promising conventional methods to generate peptides at industrial level. Thus, fermentation/enzymatic hydrolysis of dairy proteins treated with ultrasound, microwave, and high-pressure is possible to generate improved bioactive peptides at a lower cost and short time compared to only conventional applications methods.

In the dairy industry, mostly milk is used as a medium in novel technologies. So, there is a gap in understanding that either the treatment of novel technologies enhances or alters the fermentation/enzymatic hydrolysis in whole milk, fermented milk, yogurt, cheese, and other dairy products. The synergistic effect of possible novel technologies can be investigated to understand the liberation of bioactive peptides at a low cost and short time. For instance, microwave heating and ultrasound waves/HPP pressure combination could be tested to explore the effect of heat treatment and high frequency/pressure on the release of bioactive peptides from dairy proteins. As several studies reported that the applications of these novel technologies could generate lower-cost ingredients with a higher content of available amino acids for the dairy industry.

Future studies are expected to establish the actual applications of novel technologies by investigating the maximum potential of these processing technologies to comprehend their possible specificities in the proteins’ cleavage, generate novel, and known bioactive peptides, effects on specificness, and modification of amino acids in dairy proteins.

## Conclusion

It is noteworthy that milk protein hydrolysis liberates a wide variety of bioactive peptides that possess remarkable biological functions to maintain human health. The knowledge of bioactive peptides from milk and other dairy proteins and their health benefits increases with each passing day. It’s also opening new doors to exciting offers such as novel functional foods that can help manage and prevent several chronic diseases including cardiovascular diseases, diabetes, hypertension, cancer, etc.

Although the dairy industry is slow in embracing novel technologies but reported studies to depict that UAP, MAP, and HPP are innovative, environmentally friendly, and promising pretreatments to modify the profile of peptides, improve the yields of peptides, and higher liberation of bioactive peptides as compared to conventional processing technologies. Novel technologies require sustainable, environment-friendly, and highly specialized cost equipment and workers to operate the equipment. These novel processing technologies are coupled with conventional methods for unfolding, denaturing, or aggregating the milk proteins by breaking down weak molecular interactions with less or no effect on covalent bonds. The influence of pretreatments is intensified by fermentation/enzymatic hydrolysis, which results in a higher amount of liberated low molecular weight bioactive peptides, enhanced hydrolysis, and increased proteolysis of dairy proteins which ultimately increases their bioactivity. Many studies have reported the isolation of novel bioactive peptides from dairy proteins after employing novel technologies as pretreatments.

Ultrasound-assisted processing is an innovative and most efficient technology as it offers easy control, simple operation, mild operating conditions, the ability to achieve industrial amplification and production, and effective influence of auxiliary enzymatic hydrolysis. Its mechanical effects and cavitation change the protein conformation, increase the biological functionalities of enzymes, and enhance the reaction rate of enzymatic hydrolysis. Though novel technologies are innovative, environmentally friendly, and promising pretreatments, their trend is increasing and acquisitioning momentum to produce bioactive peptides.

## Author Contributions

MAM and SI: writing original draft. IH, MMANR, AR, and MSM: reviewing and editing. SI and MMANR: conceptualization and methodology. MAM, MMANR, and SAI: supervision and project administration. SAS: visualization and data curation. SAI: funding acquisition. All authors contributed to the article and approved the submitted version.

## Author Disclaimer

The contents are solely the responsibility of the authors and do not necessarily represent the official views of NIFA.

## Conflict of Interest

The authors declare that the research was conducted in the absence of any commercial or financial relationships that could be construed as a potential conflict of interest.

## Publisher’s Note

All claims expressed in this article are solely those of the authors and do not necessarily represent those of their affiliated organizations, or those of the publisher, the editors and the reviewers. Any product that may be evaluated in this article, or claim that may be made by its manufacturer, is not guaranteed or endorsed by the publisher.
